# Associations of α-linolenic acid dietary intake with very short sleep duration in adults

**DOI:** 10.3389/fpubh.2022.986424

**Published:** 2022-08-18

**Authors:** Qianning Liu, Qingsong Shan

**Affiliations:** Department of Statistics, Jiangxi University of Finance and Economics, Nanchang, China

**Keywords:** α-linolenic acid, sleep duration, odds ratio, bootstrap methods, trend analysis

## Abstract

**Objectives:**

This study aimed to investigate the association of α-linolenic acid (ALA; 18:3 ω-3) dietary intake with very short sleep duration (<5 h) in adults based on the CDC's National Health and Nutrition Examination Survey data.

**Methods:**

Multinomial logistic regression was used to explore the association of ALA intake with very short sleep. To make the estimation more robust, bootstrap methods of 1,000 replications were performed. Rolling window method was used to investigate the trend of the odds ratios of very short sleep with age. A Kruskal–Wallis test was applied to estimate the differences in the ORs of very short sleep between genders and different age groups.

**Results:**

Compared with the first tertile, the ORs of very short sleep and the corresponding 95% CIs for the second and the third tertile of dietary ALA intake in males were 0.618 (0.612, 0.624) and 0.544 (0.538, 0.551), respectively, and in females were 0.575 (0.612, 0.624) and 0.432 (0.427, 0.437). In most cases, the differences between different ages were more significant than those between different sexes. Men's very short sleep odds ratios for the second tertile of ALA intake increased linearly with age before 60.

**Conclusions:**

The risk of a very short sleep duration was negatively related to the dietary intake of ALA. The effect of ALA on very short sleep is significantly different among groups of different genders and ages.

## 1. Introduction

α-linolenic acid (ALA; 18:3 ω-3) is an essential fatty acid that cannot be synthesized by the human body and must be ingested through the diet. ALA is found in many seeds such as chia, flax, and hemp. Many plant foods (e.g., walnuts, soybeans, spinach, kale, and purslane) are also high in ALA. It additionally occurs in some seed oils, such as flaxseed and rapeseed (canola) oil, as well as some animal fats. Studies have shown that eating a diet rich in ALA and taking ALA supplements could reduce the risk of obesity (1), heart disease (2), cancer (3), and diabetes (4). ALA deficiency can lead to reduced vision, inability to walk, weakness, scaliness of skin, excessive cholesterol and inflammation, and pain in the legs (5, 6). To prevent deficiencies, between 0.2 and 0.3% of the total calories in a diet should contain ALA (5).

Short sleep problems are becoming more and more common in the United States. Over one-third of American adults reported sleeping <7 h in a 24 h period. Insufficient sleep can negatively affect energy, mood, concentration, and overall health. Short sleep duration is associated with higher mortality rates from ischemic heart disease, cancer, and stroke (7, 8), and increases the risks of hypertension, coronary heart disease, and diabetes mellitus (9–11). Dr. Thomas Roth said in Matthew Walker's book, Why We Sleep (12), “The number of people who can survive on 5 h of sleep or less without any impairment, expressed as a percent of the population, and rounded to a whole number, is zero.” Most adults need 7–9 h of sleep every night to best function, and an average of 8 h and 10 min to avoid neurobehavioral impairment. Adjusting one's diet to improve sleep is a feasible, convenient, and low-cost strategy.

ALA, eicosapentaenoic acid (EPA; 20:5 ω-3) and docosahexaenoic acid (DHA; 22:6 ω-3) are the three most important types of ω-3 fatty acids. Some ALA can be converted into EPA and then to DHA, but only in very small amounts (13, 14). Numerous studies have documented the relationship between sleep and ω-3 fatty acids (15–18). Researchers have found that DHA increased sleep efficiency and reduced sleep latency in healthy young adults (15). These results supported Yehuda animal models (19). Further, low levels of ω-3 fatty acids intake have previously been associated with sleep problems in children and obstructive sleep apnea in adults (18, 20).

Studies have shown that EPA has beneficial effects on regulating a healthy sleep cycle and reducing the risk of very long and very short sleep durations (15, 17, 21). Researchers found that DHA was beneficial for sleep in people of all ages (15–18). Although consumption of ω-3 fatty acids is known to have positive effects on sleep, prior literature has overlooked the importance of ALA, the only member of the ω-3 family considered essential. This study aims to explore the association of the dietary intake of ALA with very short sleep (<5 h). The differences in the effects of ALA on very short sleep between different genders and age groups were analyzed, respectively. The trend of ALA's effect on very short sleep with age was illustrated by rolling window.

## 2. Materials and methods

### 2.1. Participants

**Figure 1:**
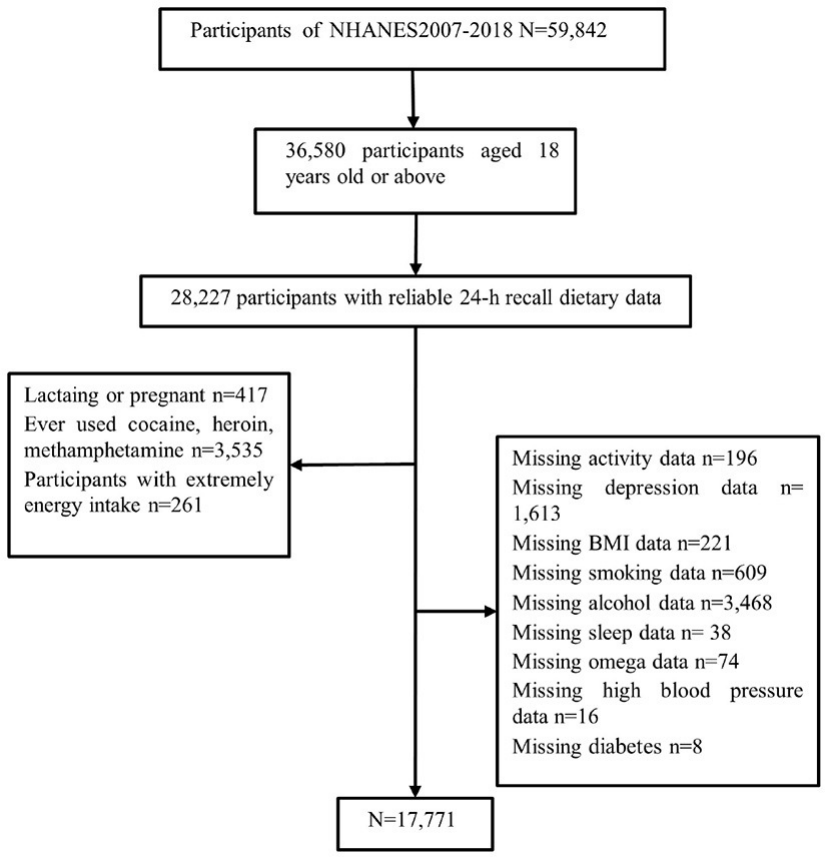
Flow chart of the screening process for the selection of eligible participants.

We studied and implemented data from the six cycles of the US National Health and Nutrition Examination Survey (NHANES; 2007–2008, 2009–2010, 2011–2012, 2013–2014, 2015–2016, and 2017–2018). The NHANES is a continuous major program that has a continually updating focus on various health and nutrition measurements to meet emerging needs; the data are released on a 2-year cycle. For a complete description of the NHANES program, see: https://www.cdc.gov/nchs/nhanes/ or previous literature (22). We deleted data from participants who lacked information about sleep-related questions, individuals under 18 years old, those using sedative-hypnotic drugs, and those with 24 h dietary recall status that did not meet the reliable or minimum standards. Many activity-related measures, including vigorous and moderate work-related activities, walking, or bicycling for transportation, and vigorous and moderate leisure-time physical activities, were summarized into a new measure named “total activity,” using the recommended MET scores. All other variables used raw data from NHANES. Figure 1 shows the detailed screening procedure. Finally, a total of 17,771 participants were involved in this study.

### 2.2. Sleep duration measurements

Data on sleep duration were based on the respondents' answers to the following question: “How much sleep do you usually get at night on weekdays or workdays?” They were further divided into very short (<5 h), short (5–7 h), normal (7–9 h), and long (≥9 h) sleep duration (23). “Normal” was set as the reference level.

### 2.3. Dietary intake of ALA

Dietary intake of ALA was obtained through two 24 h dietary recalls. In this study, the average daily intake of dietary ALA was adjusted for participants' body weights.

### 2.4. Covariates

To control for potential confounding effects, we included the following covariates: age, gender, marital status, body mass index, annual family income, race/ethnicity, educational level, smoking status, drinking status, caffeine intake, diabetes, and hypertension. As stated above, recreational and work-related physical activities were summarized into a new variable, “total activity.” The classifications of the above covariates were consistent with previous studies (24, 25) and are illustrated in Table 1.

**Table 1:** Classification and types of covariates.

**Covariates**	**Classifications**				
Age group	18–44 years	44–59 years		≥60 years	
Gender	MalesFemales				
Race/ethnicity	Mexican American	Other Hispanic	Non-Hispanic White	Non-Hispanic Black	Other races
Marital status	Married/Living with partner		Windowed/divorced/separated /Nevermarried		
Educational level	Below high school		High school		Above high school
Annual family income	< $20,000		≥$20,000		
Body mass index	<25 kg/m^2^		25– <30kg/m^2^		≥30 kg/m^2^
Smoking at least 100cigarettes in life	Yes		No		
Everhave 4/5 or more drinks every day	Yes		No		
Hypertension	Yes		No		
Diabetes	Yes		No		
Sedentary activity	Continuous variable				
Total activity score	Continuous variable				
Caffeine intake	Continuous variable				
Energy intake	Continuous variable				

### 2.5. Statistical analysis

In descriptive statistical analysis, counts (percentage) and medians (interquartile range) were used to describe qualitative and quantitative data, respectively. The Wilcoxon rank-sum test was adopted to compare continuous variables for participants with different sleep duration. The chi-square test was applied to compare qualitative variables. Body weight-adjusted intake of ALA was divided into tertiles, and the lowest tertile (tertile 1) was set as the reference. To make the estimates representative of the non-institutionalized civilian population of the United States, we weighted the analysis using the NHANES weighting guide. Multinomial logistic regression analysis was used to examine the association of sleep duration with the intake of ALA with normal sleep duration (7–9 h) as the reference. Throughout the study, the models were adjusted for sex, age, educational level, annual family income, race/ethnicity, marital status, smoking status, drinking status, caffeine intake, hypertension, diabetes, body mass index, total activity scores, and sedentary activity. Among these adjustments, the total activity scores were calculated using recommended MET scores from the following five variables: vigorous work-related activity, moderate work-related activity, walking or bicycling for transportation, vigorous leisure-time physical activity, and moderate leisure-time physical activity. Given the differences in sleep conditions between gender and age groups, we conducted a stratified analysis of sleep duration by gender and age group. We used boxplots to visualize the odds ratios (ORs) of very short sleep for male, female, and different age groups. We applied a Kruskal-Wallis test (a nonparametric statistical method) to estimate the differences in the ORs of very short sleep between genders and different age groups. To make our estimation more robust, we used bootstrap methods of 1,000 replications. Analysis was implemented using R version 4.0.3 (R Core Team, 2020) and RStudio version 1.3.1093.

### 2.6. Rolling window method

To visualize the trend of the ORs of very short sleep duration for ALA intake with age, we conducted the following analysis: To establish a multinomial logistic regression model, we took each age from 18 to 70 years as the starting age and the subsequent 15 years of rolling window data. For example, when the starting age was 18 years, we selected individuals between 18 and 33 years as the analysis object and calculated the regression coefficient of ALA intake in the multinomial logistic regression model. When the starting age was 19 years, we selected participants aged 19–34 as the analysis object, and so on.

## 3. Results

Table 2 shows the characteristics of the study participants by sleep durations. Among the 17,771 participants, the ratio of men to women was approximately 1:1. The proportion of very short sleepers was about 4.63%. Moreover, compared with normal sleepers, they tended to be older, non-Hispanic black, have lower educational level, widowed/living-alone, lower family income, higher BMI index, hypertension, diabetes, more alcohol drinks, less sedentary activity, fewer activity scores, and depression. The median dietary intake of ALA and the corresponding 95% CI were 1.34 and (0.86, 2.00) mg/day, respectively, for participants with very short sleep, compared to 1.68 (1.13, 2.43) mg/day for those with normal sleep. It indicated that participants with very short sleep had significantly lower levels of ALA dietary intake.

**Table 2:** Sample characteristics by sleep durations, NHANES 2007–2018 (*N* = 17,771).

**Characteristic**	**7– <9 h, *N* = 9,644^a^**	** <5 h, *N* = 822^a^**	**5– <7 h, *N* = 5,176^a^**	**≥9 h, *N* = 2,129^a^**	***p*-value^b^**
Gender (%)					<0.001
Male	4,863 (50%)	385 (47%)	2,726 (53%)	989 (46%)	
Female	4,781 (50%)	437 (53%)	2,450 (47%)	1,140 (54%)	
Age (year) (%)					<0.001
18-44	3,920 (41%)	291 (35%)	2,136 (41%)	775 (36%)	
44-59	2,159 (22%)	237 (29%)	1,356 (26%)	332 (16%)	
60-	3,565 (37%)	294 (36%)	1,684 (33%)	1,022 (48%)	
Race/ethnicity (%)					<0.001
Mexican American	1,410 (15%)	81 (9.9%)	666 (13%)	293 (14%)	
Other hispanic	909 (9.4%)	91 (11%)	597 (12%)	198 (9.3%)	
Non-hispanic white	4,608 (48%)	284 (35%)	1,958 (38%)	1,014 (48%)	
Non-hispanic black	1,645 (17%)	302 (37%)	1,474 (28%)	424 (20%)	
Other race	1,072 (11%)	64 (7.8%)	481 (9.3%)	200 (9.4%)	
Educational level (%)					<0.001
Above	5,684 (60%)	360 (44%)	2,847 (56%)	1,050 (52%)	
Below	1,813 (19%)	230 (28%)	1,062 (21%)	467 (23%)	
High school	1,986 (21%)	225 (28%)	1,198 (23%)	514 (25%)	
Unknown	161	7	69	98	
Marital status (%)					<0.001
Married/Cohabitation	6,052 (64%)	428 (53%)	3,009 (59%)	1,168 (57%)	
Windowed/Living alone	3,433 (36%)	387 (47%)	2,099 (41%)	865 (43%)	
Unknown	159	7	68	96	
Annual family income (%)					<0.001
<20,000	1,753 (19%)	269 (34%)	1,083 (22%)	549 (27%)	
≥20,000	7,445 (81%)	520 (66%)	3,877 (78%)	1,485 (73%)	
Unknown	446	33	216	95	
Body mass index (%)					<0.001
<25	2,865 (30%)	185 (23%)	1,285 (25%)	630 (30%)	
Between	3,257 (34%)	221 (27%)	1,714 (33%)	674 (32%)	
Above30	3,522 (37%)	416 (51%)	2,177 (42%)	825 (39%)	
Caffeine intake (mg/d)	104 (34, 204)	86 (22, 190)	102 (32, 206)	88 (24, 182)	<0.001
Total energy (kcal/day)	1,917 (1,492, 2,427)	1,735 (1,320, 2,300)	1,910 (1,467, 2,477)	1,822 (1,402, 2,298)	<0.001
Hypertension (%)					<0.001
Yes	3,355 (35%)	387 (47%)	1,983 (38%)	880 (41%)	
No	6,289 (65%)	435 (53%)	3,193 (62%)	1,249 (59%)	
Diabetes (%)					<0.001
Yes	1,166 (12%)	145 (18%)	713 (14%)	353 (17%)	
No	8,258 (86%)	657 (80%)	4,324 (84%)	1,729 (81%)	
Borderline	220 (2.3%)	20 (2.4%)	139 (2.7%)	47 (2.2%)	
Smoked at least 100 cigarettes in life (%)					<0.001
Yes	4,035 (42%)	450 (55%)	2,311 (45%)	962 (45%)	
No	5,609 (58%)	372 (45%)	2,865 (55%)	1,167 (55%)	
Ever have 4/5 or more drinks every day (%)					<0.001
Yes	1,137 (12%)	165 (20%)	715 (14%)	299 (14%)	
No	8,507 (88%)	657 (80%)	4,461 (86%)	1,830 (86%)	
Sedentary activity (minutes/day)	360 (180, 480)	300 (180, 480)	300 (180, 480)	360 (240, 480)	0.007
Total activity (per week)	880 (0, 2,760)	360 (0, 2,640)	840 (0, 2,880)	540 (0, 2,160)	<0.001
Unknown	1,225	146	794	230	
Depression					<0.001
Normal	9,112 (94%)	599 (73%)	4,710 (91%)	1,931 (91%)	
Depression	532 (5.5%)	223 (27%)	466 (9.0%)	198 (9.3%)	
α-Linolenic Acid (mg/pound/day)	1.68 (1.13, 2.43)	1.34 (0.86, 2.00)	1.58 (1.05, 2.30)	1.62 (1.09, 2.38)	<0.001

^a^*n* (%); Median (IQR).

^b^Pearson's Chi-squared test; Kruskal–Wallis rank sum test.

**Figure 2:**
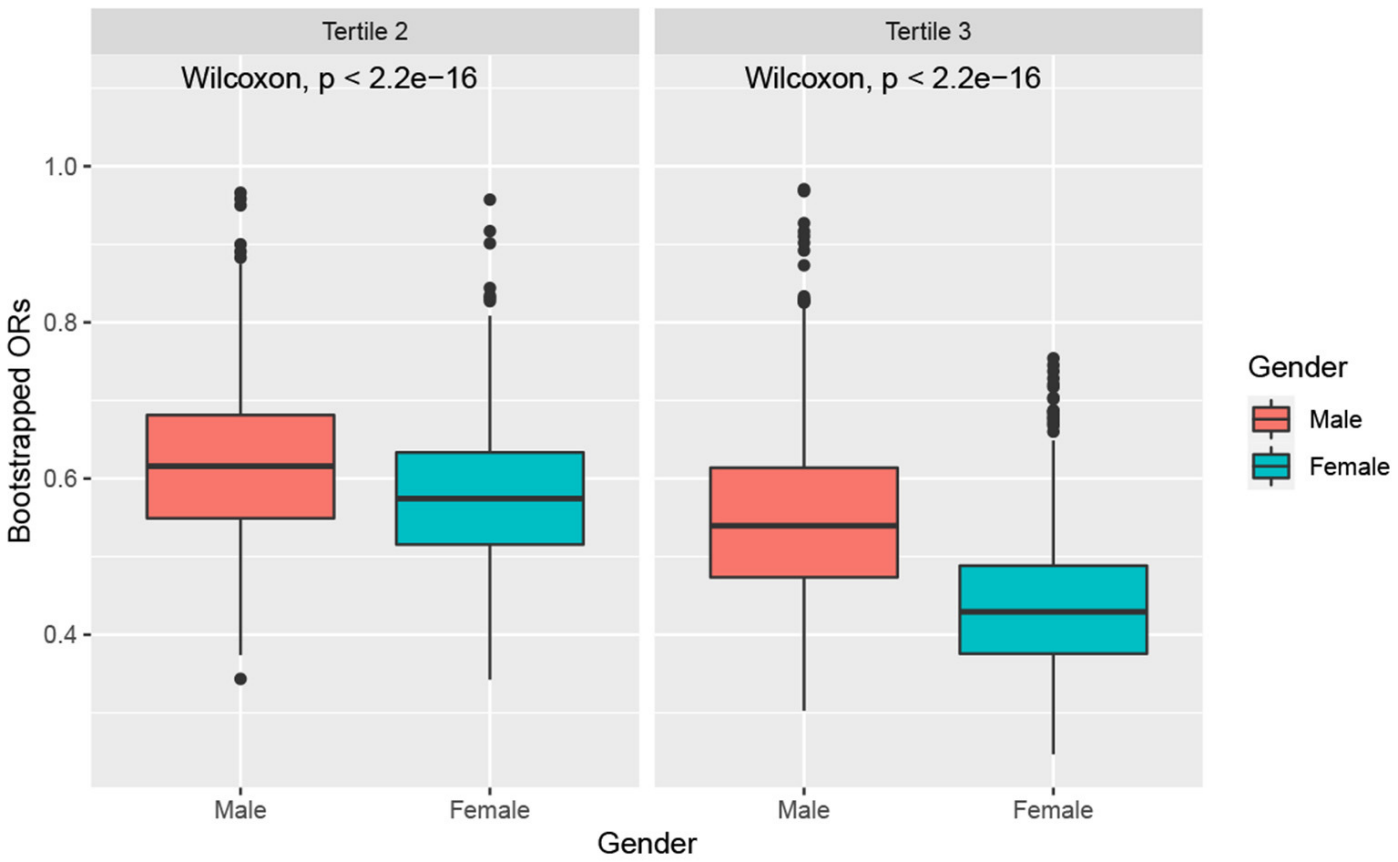
Boxplot of bootstraped ORs by gender. The model was adjusted for age, race, educational level, marital status, annual family income, BMI, caffeine intake, total energy intake, hypertension, diabetes, smoking status, drinking status, sedentary activity, and total activity per week.

Figure 2 illustrates the boxplots of the bootstrapped ORs of very short sleep for the second and highest tertiles of ALA obtained from the bootstrapped samples of men and women. We used the Kruskal–Wallis test to check whether the ORs of very short sleep for different genders had the same median. Figure 2 shows that the ORs of different genders were significantly different for both the second and highest tertiles of ALA dietary intake. In general, the ORs of very short sleep duration for the dietary intakes of ALA in women were lower than those in men, which was more pronounced for the third tertile of ALA intake.

**Figure 3:**
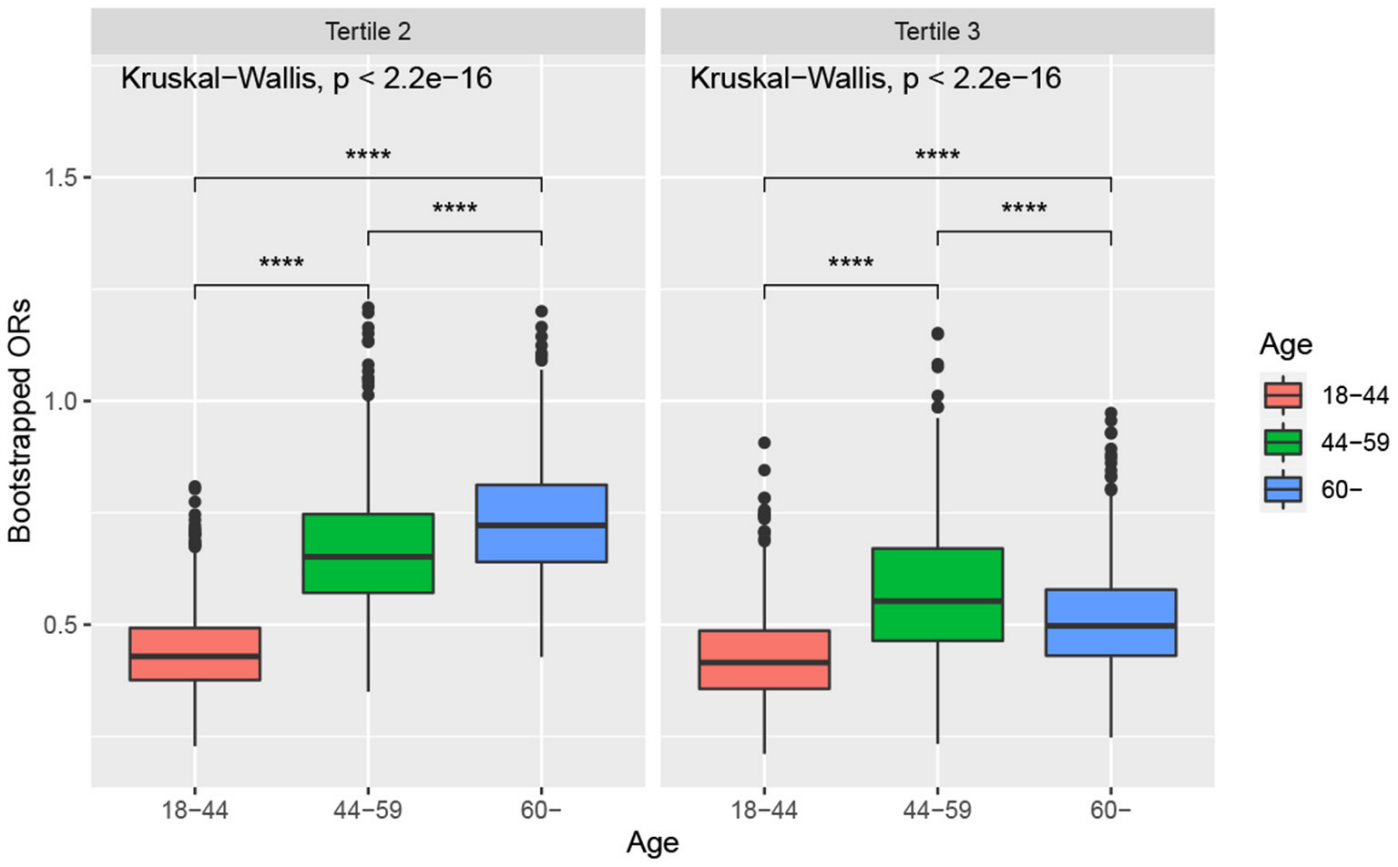
Boxplot of bootstrapped ORs by age. The model was adjusted for sex, race, educational level, marital status, annual family income, BMI, caffeine intake, total energy intake, hypertension, diabetes, smoking status, drinking status, sedentary activity, and total activity per week. Note: * *p* < 0.05, ** *p* < 0.01, *** *p* < 0.001, **** *p* < 0.0001.

Figure 3 shows the boxplots of the bootstrapped ORs of very short sleep for ALA intake in different age groups. For the second tertile of dietary intakes of ALA, the difference in the ORs between people aged 45–59 and those over 60 years was not apparent. In contrast, the differences in other pairwise comparisons were apparent. The ORs of very short sleep for both the second and the highest tertiles of dietary intakes of ALA in young people ages 18–44 were relatively low. People 60 years or older were more sensitive to high levels of dietary intake of ALA.

We built regression models to investigate the effect of ALA dietary intake on very short sleep in men and women. To make the estimations more robust, we used the bootstrap method. We resampled the male and female groups 1,000 times each. Further, we performed multinomial logistic regressions for each subsample. We recorded the regression coefficients (i.e., ORs of very short sleep for the second and third tertile of dietary ALA intake) with the first tertile of ALA intake being the reference. As shown in Table 3, dietary ALA intake was negatively related to very short sleep risk in both women and men, and the ORs in women were lower than those in men for both the second and the third tertiles of ALA intake. To estimate the differences in the effect of ALA dietary intake on very short sleep between men and women, we used the bootstrap method again. Specifically, we resampled each gender group 1,000 times and calculated the regression equations' ALA intake coefficients (i.e., OR). Further, we used the ORs' distribution of very short sleep for both the second and third tertiles of ALA dietary intake for each group to calculate the difference and its 95% CI between any two adjacent groups. The difference in the ORs of very short sleep for the third tertile of ALA intake was significantly higher than the second, which means that by increasing the dietary intake of ALA, women can improve very short sleep more effectively than men.

**Table 3:** Bootstrapped odds ratios (95% confidence intervals) of very short sleep (reference, 7–9 h/night) across tertiles of body weight-adjusted dietary ALA intake and differences between odds (95% bootstrapped percentile confidence intervals), stratified by gender, NHANES 2007–2018.

	**Male**	**Difference between genders**	**Female**
**Very short sleep duration (<5 h/night)**			
≤ 1.27	1.00 (ref)		1.00 (ref)
(1.27, 2.08]	0.602**1*	0.018**1*	0.583**1*
	(0.596, 0.608)	(0.01, 0.027)	(0.578, 0.588)
>2.08	0.533**1*	0.087**1*	0.445**1*
	(0.527, 0.539)	(0.079, 0.095)	(0.439, 0.45)

^+^ p < 0.1,

^*^ p < 0.05,

^**^ p < 0.01,

^***^ p < 0.001.

Calculated using multinomial logistic regression models, adjusted for age, race, educational level, marital status, annual family income, BMI, caffeine intake, total energy intake, hypertension, diabetes, smoking status, drinking status, sedentary activity, and total activity per week.

Similarly, we explored the impact of ALA dietary intake on very short sleep for people of different ages using three age groups. Table 4 lists the differences between the adjacent two age groups, denoted as Difference 1 and Difference 2. Difference 1 represents the difference between the 18–44 and 45–60 years groups; Difference 2 is the difference between the 44–60 and 60+ years groups. In most cases, the differences between different ages were more significant than those between different sexes. The smallest difference in the ORs of very short sleep for adjacent age groups was the difference between the 44–60 and 60+ years groups for the third tertile of ALA dietary intake. The difference, −0.220 (−0.230, −0.210), between the 18–44 and 44–60 years groups for the second tertile of ALA dietary intake was the largest.

**Table 4:** Bootstrapped odds ratios (95% confidence intervals) of very short sleep (reference, 7–9 h/night) across tertiles of body weight-adjusted dietary ALA intake and differences between adjacent age groups (95% bootstrapped percentile confidence intervals), stratified by age, NHANES 2007–2018.

	**18–44 years**	**Difference 1**	**44–60 years**	**Difference 2**	**60+ years**
**Very short sleep duration (<5 h/night)**					
≤ 1.27	1.00 (ref)		1.00 (ref)		1.00 (ref)
(1.27, 2.08]	0.441**1*	−0.203**1*	0.648**1*	−0.091**1*	0.737**1*
	(0.435, 0.447)	(−0.213, −0.193)	(0.639, 0.657)	(−0.102, −0.079)	(0.73, 0.745)
>2.08	0.442**1*	−0.1**1*	0.546**1*	0.018**	0.525**1*
	(0.436, 0.449)	(−0.111, −0.089)	(0.537, 0.555)	(0.007, 0.03)	(0.517, 0.532)

^+^ p < 0.1,

^*^ p < 0.05,

^**^ p < 0.01,

^***^ p < 0.001.

Calculated using multinomial logistic regression models, adjusted for sex, race, educational level, marital status, annual family income, BMI, caffeine intake, total energy intake, hypertension, diabetes, smoking status, drinking status, sedentary activity, and total activity per week.

**Figure 4:**
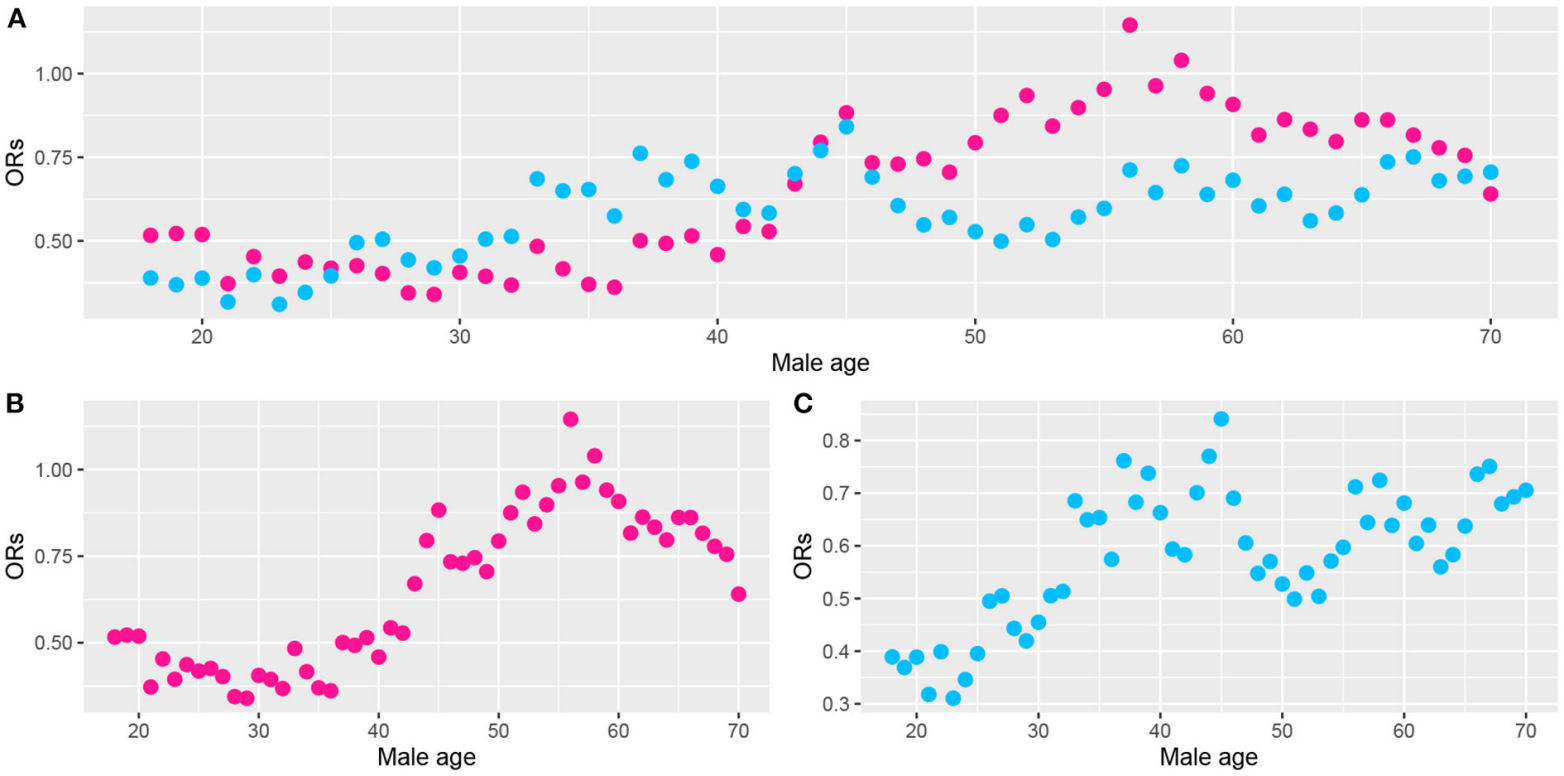
Scatter plot of the odds ratios (ORs) calculated from male data (aged from 18 to 70) in a rolling window with a 15-year time interval. **(B)** ORs of men's very short sleep for the second tertile of ALA intake. **(C)** ORs for the third tertile. **(A)** ORs for the second (pink) and the third tertile (skyblue) of ALA intake. Models were adjusted for race, educational level, marital status, annual family income, BMI, caffeine intake, total energy intake, hypertension, diabetes, smoking status, drinking status, sedentary activity, and total activity per week.

**Figure 5:**
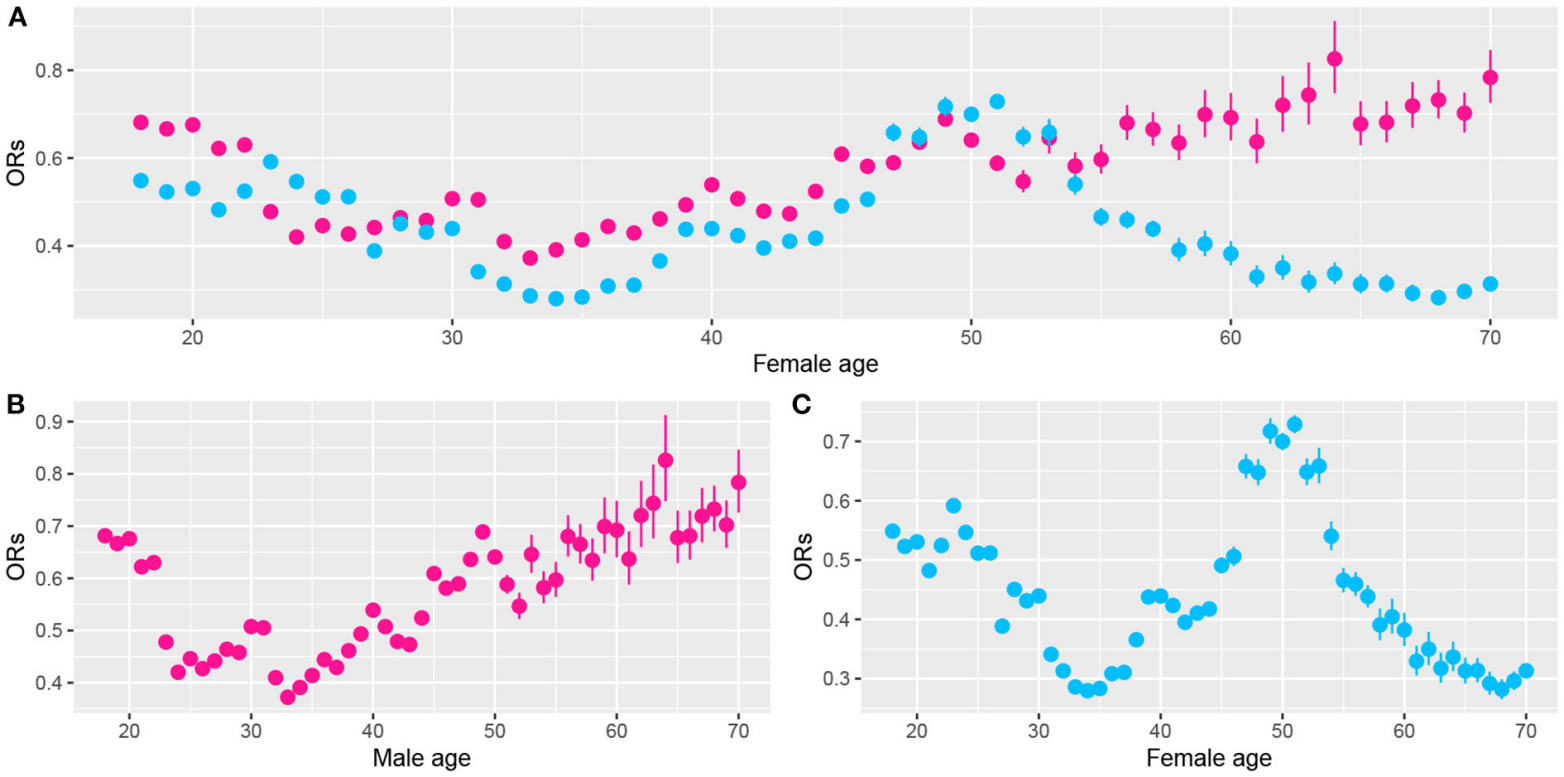
Scatter plot of the odds ratios (ORs) calculated from female data (aged from 18 to 70) in a rolling window with a 15-year time interval. **(B)** ORs of women's very short sleep for the second tertile of ALA intake. **(C)** ORs for the third tertile of ALA intake. **(A)** ORs for the second (pink) and the third tertile (sky-blue) of ALA intake. Models were adjusted for race, educational level, marital status, annual family income, BMI, caffeine intake, total energy intake, hypertension, diabetes, smoking status, drinking status, sedentary activity, and total activity per week.

We noted statistically significant differences in the effects of ALA dietary intake on very short sleep duration for different genders and ages. To further analyze the trend of the ORs of very short sleep for the second and the highest tertiles of dietary ALA intake with age in different sexes, we conducted the rolling window method to visualize the trend of the ORs of very short sleep duration for ALA intake with age. Figures 4, 5 show the regression coefficients, that is, the ORs of very short sleep for the second and the third tertiles of ALA intake in women and men with age. Figure 4 shows that the ORs of men's very short sleep for the second tertile of ALA intake increased linearly with age before the age of 60 and then decreased. Before age 45, there was no significant difference in the ORs of very short sleep duration in men for the second and the third tertiles of ALA intake. From age 45, the ORs in men for the third tertile of ALA intake were lower than those for the second tertile. For women under 55 years of age (Figure 5), the ORs of very short sleep for the second tertile of ALA intake were not significantly different from those for the third tertile. The ORs for the highest tertile were significantly lower than those for the second.

**Figure 6:**
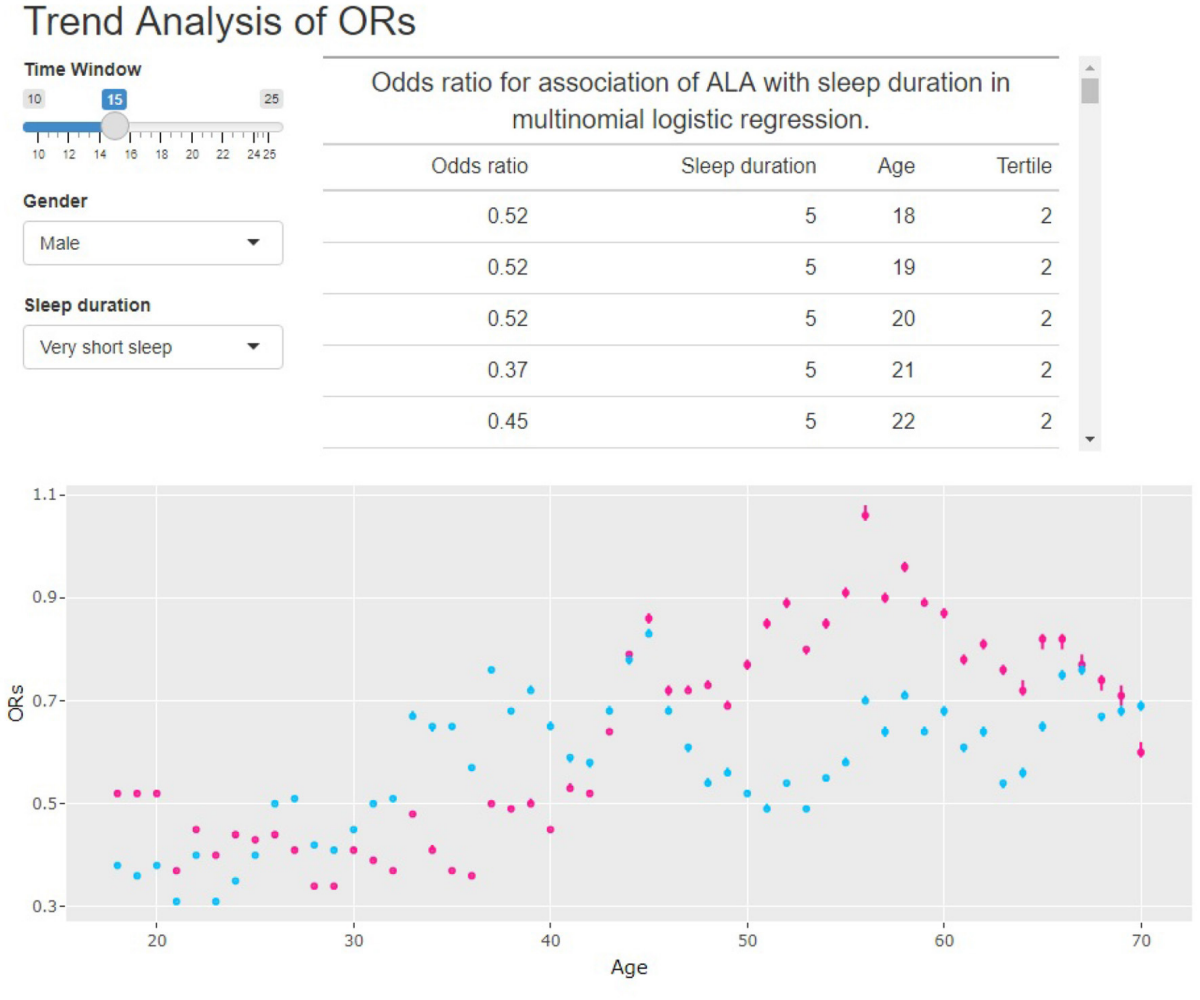
Interface of the Shiny APP.

In this paper, the rolling window data analysis used 15 years as the window width. We noticed that even though 15 was not the only choice, a short window width would lead to unstable results. On the other hand, a long window width would increase the calculation time. To illustrate this, we built a Shiny app at http://shiny.statlearning.com.cn/TrendAnalysisALA/. Figure 6 shows the interface of the app. It shows the ORs trend of tertile 2 (pink) and tertile 3 (sky blue) of ALA intake for any given window width and gender.

## 4. Discussion

To our knowledge, this is the first study that uses the bootstrap method to estimate the differences in the impact of ALA dietary intake on very short sleep duration between different genders and age groups. We proposed a new method to visualize the influence of ALA dietary intake on the risk of short sleep duration in men and women with age. Moreover, this is the first study based on a large, nationally representative sample of American adults that explores the association of dietary ALA intake with sleep disorders and very short sleep. Unlike previous studies (24, 26), we used body weight instead of energy intake to adjust dietary ALA intake. Our analysis results were statistically more significant; therefore, they were more reliable for application. In addition, since body weight is easier to measure than energy intake, it was more convenient for individuals to apply our analysis results to adjust their daily dietary ALA intake according to their body weights. Our estimations were based on the bootstrap method, making them more robust than previous studies.

Our analysis indicated that the dietary ALA intake was generally negatively correlated with the risk of sleep disorders and very short sleep duration. Researchers reported that a mixture of linoleic and ALA with a ratio of 4:1 was the most effective in improving sleep (19). Prior study also showed the effects of a mixture of ALA and linoleic acid on behavioral variables related to anxiety (mood, poor sleep, appetite, fatigue, mental concentration, and academic organization) (27). Our findings are consistent with these previous conclusions. We also found that the ORs for ALA intake were generally lower and more significant in the sleep duration analysis than those in the sleep disorder analysis. This result might be because the self-reported binary response data on whether there is a sleep disorder is not as accurate as the self-reported sleep duration data. This is because the answers to questions about sleep disorders mainly emphasized whether a sleep disorder had ever been experienced. However, the responses to sleep duration were current objective sleep measurements.

We concluded that dietary ALA intake was negatively related to very short sleep risk. The following reasons may explain this. First, the high serum ALA and linoleic acid levels in free fatty acids were linked to decreased odds of depressive symptoms in Japanese adults, supporting a protective role of ALA and linoleic acid against depression (28). In a longitudinal study of women, supplementation of ALA in the diet reduced symptoms of depression (29). Some of the core features of depression include sleep problems. Second, oral consumption of ALA increases serum brain-derived neurotrophic factor (BDNF) levels in healthy adult humans (30). BDNF has an essential role in cognitive function and has been linked to clinical insomnia (31). Third, a previous study showed that dietary ALA and linoleic acid elevated melatonin activity in the hepatoma of rats (32). Melatonin is a “sleep hormone” that can help people fall asleep faster (33, 34). Many of these studies reported significant improvements in sleep quality (35). Fourth, poor sleep is related to inflammation (36, 37), and ALA can be used as an effective anti-inflammatory agent (38–40). Fifth, 5-hydroxytryptamine (5-HT) receptor 1A and 5-HT receptor 2A levels were enhanced by ALA-rich diets (41). 5-HT was considered as an important substance for sleep preparation, triggering and maintenance (42).

We found significant differences in the impact of ALA dietary intake on the risk of very short sleep duration for different genders and age groups. The trend analysis showed that the pattern for men by age differs from that of women, which may be related to sex hormones. Compared to men, women use a smaller proportion of α-linoleic acid as the substrate for β-oxidation, and a higher proportion is converted into long-chain fatty acids ω-3, possibly due to the regulation of estrogen (43). Previous studies have shown that ω-3 fatty acids improved sleep problems (24, 44). Furthermore, we found that higher dietary intake of ALA was associated with significantly lower ORs of very short sleep duration for older women and men and middle-aged men. The aging process, along with lifestyle changes and multiple comorbidities, can bring about many changes in sleep (45). Hormonal shifts during menstrual cycles and menopause may also play a role. During menopause, night sweats and hot flashes often disrupt sleep. ALA has significant effects on postmenopausal symptoms such as hot flashes, insomnia, and headaches, as well as balancing sex hormone levels in women with polycystic ovary syndrome (46).

Our results suggest that adequate dietary ALA intake may significantly improve very short sleep problem in middle-aged men, as well as older women and men. Our results also provide a theoretical reference for how to improve the issue of very short sleep duration through diet. Our findings can contribute theoretically and practically to the scientific fields of diet and sleep.

In 2002, the Food and Nutrition Board of the U.S. Institute of Medicine established adequate intake levels for 1.6 and 1.1 g/day ALA intake in male and female adults, respectively (47). In 2009, The European Food Safety Authority published its recommendations for 2 g/day ALA intake (48). Dietary intake of ALA intake should take these limitations into account.

Our research has several limitations and we need to interpret the conclusions with caution. First, the methods used in this paper, such as bootstrap and setting the rolling window to 15 years, can control the influence of confounding factors to a certain extent. However, due to the availability of data, some confounding factors, such as relative changes in sleep duration, use of sleep medication, etc., are not taken into account in the model. Second, the data on sleep disorders and durations were collected based on self-reported sleep wellness in personal interviews rather than physical examinations or medical chart reviews. Self-reported sleep duration is commonly used in population health monitoring studies because it offers several advantages (e.g., non-invasive, inexpensive, and logistically easy to manage for large individual samples). However, self-reported sleep duration usually overestimates actual sleep duration (49); it may also contain recall and reporting biases and may not reflect the actual sleep conditions. Third, participants' primary care providers may not have diagnosed some sleep disorders, so the reported prevalence of disorders may be lower than the objective prevalence. Finally, although the correlations between sleep disorders, very short sleep durations, and many covariates were explained as a factor in this study, our study was cross-sectional and could not confirm causation. Further longitudinal studies should validate the causes behind these associations.

## 5. Conclusions

ALA is considered a fundamental member of the ω-3 family, but its importance in regulating sleep is overlooked. Our study provides evidence supporting the role of ALA in improving very short sleep. Further, we found that the effects of ALA on sleep are age- and gender-dependent. Our findings can be used in dietary ALA intake guidelines to regulate very short sleep problems in different gender and age groups. Our use of the bootstrap method resulted in more robust and reliable findings. To our knowledge, no literature used the bootstrap method to investigate the relationship between sleep and specific dietary factors. Moreover, we built a Shiny app at http://shiny.statlearning.com.cn/TrendAnalysisALA/ for comparing the trends of ORs with different window widths.

## Data availability statement

Publicly available datasets were analyzed in this study. This data can be found at: https://www.cdc.gov/nchs/nhanes/.

## Author contributions

QL: study design, data analysis, and manuscript preparation. QS: methodology, software, and validation. All authors contributed to the article and approved the submitted version.

## Conflict of interest

The authors declare that the research was conducted in the absence of any commercial or financial relationships that could be construed as a potential conflict of interest.

## Publisher's note

All claims expressed in this article are solely those of the authors and do not necessarily represent those of their affiliated organizations, or those of the publisher, the editors and the reviewers. Any product that may be evaluated in this article, or claim that may be made by its manufacturer, is not guaranteed or endorsed by the publisher.
